# Citrulline supplementation in postmenopausal women: a systematic review of vascular, muscular, and metabolic effects

**DOI:** 10.1186/s12905-026-04277-6

**Published:** 2026-01-26

**Authors:** Hossein Bahari, Elmira Ramezani, Mahsa Malekahmadi

**Affiliations:** 1 Department of Nutrition, Faculty of Medicine, Mashhad University of Medical Sciences, Mashhad, Iran; 2 Department of Nutrition, Faculty of Public Health, Iran University of Medical Sciences, Tehran, Iran; 3 Imam Khomeini Hospital Complex, Tehran University of Medical Sciences, Tehran, Iran

**Keywords:** Citrulline, Postmenopausal women, Cardiovascular health, Watermelon, Nitric oxide

## Abstract

**Background:**

Postmenopausal women are at increased risk of developing cardiovascular, muscular, and metabolic dysfunction due to hormonal changes associated with aging. Citrulline, a non-essential amino acid and precursor to nitric oxide, has gained interest as a potential dietary supplement for improving vascular health, muscle function, and metabolic parameters in this population.

**Objective:**

This systematic review aims to evaluate the effects of citrulline supplementation, administered directly or via watermelon products, on cardiovascular, muscular, and metabolic outcomes in postmenopausal women.

**Methods:**

A comprehensive literature search was conducted using PubMed, Web of Science, and Scopus databases to identify randomized controlled trials (RCTs) investigating citrulline supplementation in postmenopausal women. Studies were included if they reported outcomes related to blood pressure, arterial stiffness, endothelial function, muscle strength or mass, metabolic parameters, and safety. Study quality was assessed using the Cochrane Risk of Bias Tool 2. Due to heterogeneity in study designs and reported outcomes, results were synthesized narratively.

**Results:**

Twelve RCTs involving 360 postmenopausal women were included. all conducted in the United States, with study durations ranging from 4 to 8 weeks and participant ages between 50 and 75 years. Seven studies reported blood pressure outcomes, with most showing reductions in systolic blood pressure and mean arterial pressure. Five studies examined arterial stiffness, with mixed findings on pulse wave velocity and augmentation index. Four studies assessed endothelial function, two of which demonstrated significant improvements in flow-mediated dilation. Muscle function outcomes were investigated in two studies, suggesting improvements only when citrulline was combined with resistance training. Six studies assessed metabolic parameters, with no consistent effects observed on body weight, glucose, insulin, or lipid profiles. Across all studies, no adverse effects related to citrulline supplementation were reported.

**Conclusion:**

Citrulline supplementation may offer benefits for blood pressure regulation (up to 9 mmHg SBP reduction in some studies) in hypertensive postmenopausal women, but evidence for arterial stiffness, endothelial function, and metabolic outcomes remains inconsistent. Further large-scale studies are needed before clinical recommendations can be made.

**Supplementary Information:**

The online version contains supplementary material available at 10.1186/s12905-026-04277-6.

## Introduction

The postmenopausal period represents a significant transition in a woman’s life, marked by the permanent cessation of ovarian function and a dramatic decline in circulating estrogen levels [1]. This hormonal shift is associated with numerous physiological changes that predispose women to a variety of health challenges, including increased cardiovascular risk, unfavorable body composition changes (such as increased fat mass and decreased lean mass), impaired metabolic function, and vascular endothelial dysfunction [2]. Collectively, these changes contribute to the elevated incidence of hypertension, atherosclerosis, insulin resistance, and sarcopenia in postmenopausal women, which in turn increases their risk of major cardiovascular events, functional disability, reduced quality of life, and mortality compared to premenopausal counterparts [2, 3].

As these health risks escalate with age, identifying safe, accessible, and effective interventions becomes increasingly important. Lifestyle strategies, such as exercise and dietary modifications, have long been advocated for mitigating postmenopausal health decline [4, 5]. Beyond lifestyle strategies, specific nutritional supplements have garnered attention for mitigating postmenopausal health decline. Among these, citrulline has emerged as a promising candidate [6, 7].

Citrulline plays a crucial role in the urea cycle and serves as a precursor to L-arginine, which is subsequently converted into nitric oxide (NO), a key vasodilator involved in maintaining vascular tone, endothelial function, and blood pressure regulation [8]. Unlike L-arginine, citrulline bypasses hepatic metabolism and exhibits superior bioavailability, making it a more effective agent for increasing systemic arginine and NO levels [9, 10]. In addition to its cardiovascular benefits, citrulline may improve skeletal muscle function and oxygen delivery by enhancing blood flow and reducing muscle fatigue, which could be particularly advantageous for postmenopausal women at risk of muscle loss and frailty [6, 11–13].

Recent randomized controlled trials (RCTs) have explored the effects of citrulline supplementation, either alone or in combination with other interventions, on various health outcomes in postmenopausal women [12, 14, 15]. These studies have assessed a broad range of parameters, including blood pressure, arterial stiffness, endothelial function, muscle strength, metabolic biomarkers, and inflammatory markers [16–19]. However, findings across studies remain inconsistent, and its overall efficacy and safety profile are unclear.

Given the growing interest in citrulline as a non-pharmacological intervention with potential benefits across multiple health domains in postmenopausal women, a comprehensive synthesis of the evidence is warranted. This systematic review therefore aims to critically evaluate RCTs investigating the effects of citrulline supplementation, either in pure form or from natural sources like watermelon, on cardiovascular, muscular, and metabolic outcomes. By analyzing and integrating findings from these trials, we seek to elucidate citrulline’s therapeutic potential and inform future research and clinical applications for this demographic. To our knowledge, this represents the first systematic review to comprehensively assess citrulline supplementation across these three key health domains specifically in postmenopausal women.

## Methods

This systematic review was conducted to evaluate the effects of citrulline supplementation, administered directly or via watermelon products, on postmenopausal women. The protocol for this systematic review was prospectively registered with the International Prospective Register of Systematic Reviews (PROSPERO) under registration number CRD420251169359 (https://www.crd.york.ac.uk/prospero/display_record.php?RecordID=1169359). The review was performed in accordance with the PRISMA (Preferred Reporting Items for Systematic Reviews and Meta-Analyses) guidelines [20]. A completed PRISMA checklist is provided in Supplementary Material.

### Search strategy

A comprehensive literature search was conducted from database inception to May 2025 to identify relevant RCTs investigating the effects of citrulline supplementation in postmenopausal women. The search was performed using the following electronic databases: PubMed, Web of Science, and Scopus. The complete search strategy with Boolean operators for each database is provided in Supplementary Table 1. Additionally, reference lists of included studies and relevant reviews were manually searched, but no additional eligible studies were identified. No language restrictions were applied to the search.

### Study selection

Two independent reviewers (H.B. and E.R.) screened all retrieved studies in two stages: (1) Title and Abstract Screening: Studies were first screened based on their titles and abstracts. (2) Full-Text Screening: The full-text articles of potentially eligible studies were assessed for inclusion based on the eligibility criteria outlined above. Any disagreements during the study selection process were resolved through discussion or by consulting a third reviewer (M.M.).

### Inclusion and exclusion criteria

Eligibility criteria were defined using the PICOS framework: Population (postmenopausal women), Intervention (citrulline supplementation), Comparison (placebo/no intervention), Outcomes (cardiovascular, muscular, metabolic measures), Study design (RCTs). Studies were included if they met the following criteria: (1) RCTs involving postmenopausal women. (2) Investigation of citrulline supplementation, either in its pure form or derived from natural sources (e.g., watermelon). (3) Reporting of outcomes related to cardiovascular health (e.g., blood pressure, arterial stiffness, endothelial function), muscle function (e.g., strength, mass), or metabolic parameters (e.g., glucose, insulin, lipid profiles). (4) Studies published in peer-reviewed journals.

Exclusion criteria included non-RCT designs, studies involving premenopausal women or other populations, and studies not reporting relevant outcomes (Supplementary Table 2).

### Data extraction

Data were independently extracted by two reviewers (H.B. and E.R.) using a standardized data extraction form. The extracted information included:

- Study characteristics (author, year, country, study design).
- Participant details (sample size, age, body mass index (BMI)).
- Intervention details (type of citrulline supplementation, dose, duration, control group).
- Primary and secondary outcomes.

### Quality assessment

The risk of bias for each included study was assessed using the Cochrane Risk of Bias Tool for Randomized Trials (RoB 2) [21]. Two reviewers (H.B. and E.R.) independently assessed risk of bias. Any disagreements were resolved through discussion or by consultation with a third reviewer (M.M.). The tool evaluates bias arising from the randomization process, deviations from intended interventions, missing outcome data, measurement of outcomes, and selection of reported results. Each domain was rated as “low risk,” “high risk,” or “unclear risk.” The overall risk of bias for each study was categorized as low, some concerns, or high.

### Data synthesis

Due to heterogeneity in interventions, outcome measures, and participant characteristics, quantitative meta-analysis was not feasible. Instead, the results were synthesized narratively [22], focusing on the effects of citrulline supplementation on cardiovascular, muscular, and metabolic outcomes in postmenopausal women. Key findings were summarized and discussed in the context of the existing literature.

## Results

### Study characteristics

**Fig. 1:**
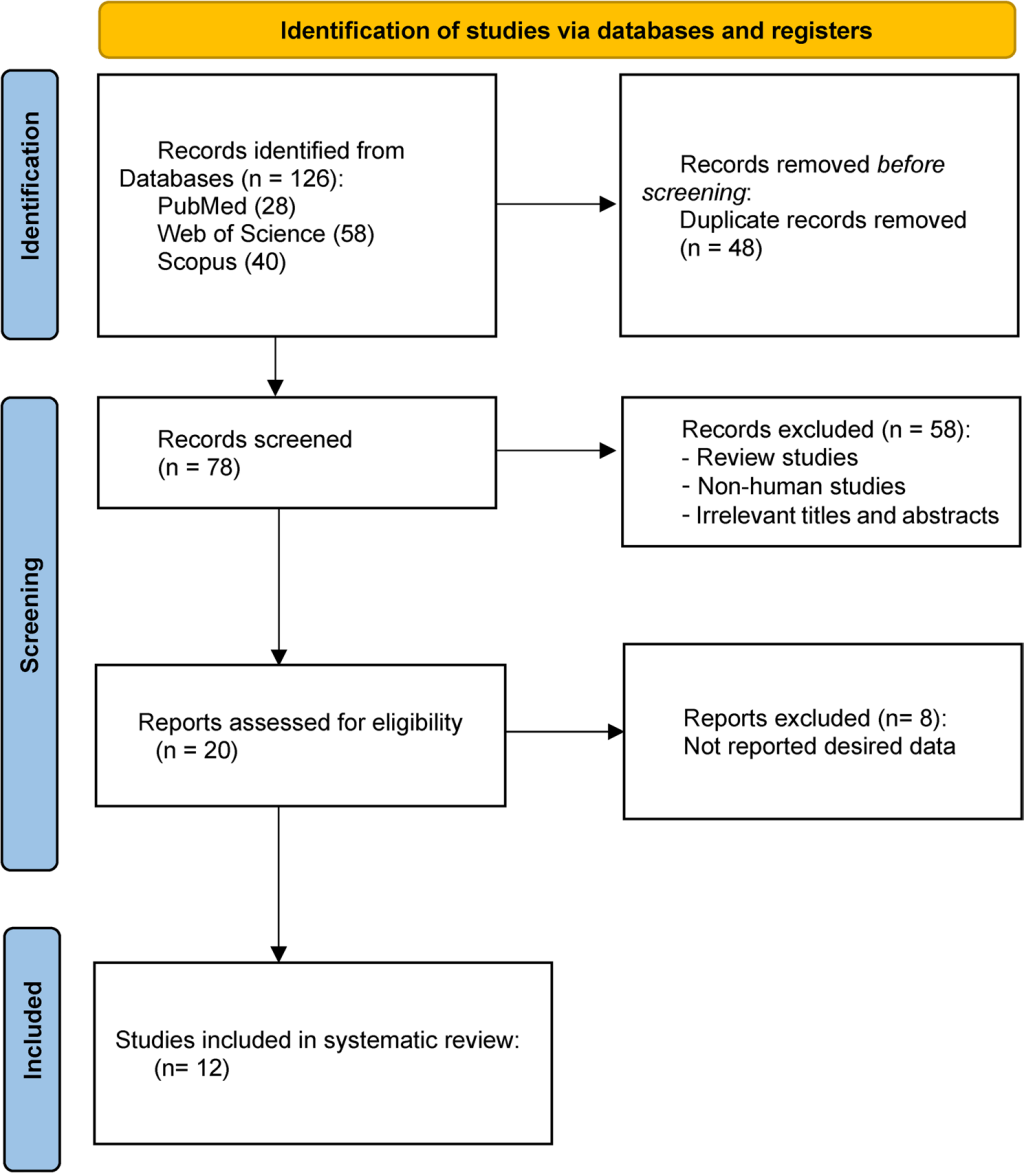
PRISMA flow chart of study selection process in the systematic review

A total of 126 articles were found in the primary search in PubMed, Web of Science, and Scopus databases that investigated the effect of citrulline on postmenopausal women from different aspects. 48 duplicate articles were removed, and 66 articles were eliminated based on the inclusion criteria (Fig. 1). This review included 12 clinical trial studies, focusing on citrulline supplementation or in combination with various types of exercises [12, 14, 18].

We organized the studies in a comprehensive table according to country, study design, participants, duration, author’s name, publication year, intervention, and outcomes of each study.

In Table 1, all clinical trials investigating the effect of citrulline on postmenopausal women are listed. Two out of twelve eligible studies were conducted in 2024 [14, 16], the other studies were conducted in 2023 [18, 19], 2022 [12, 15], 2021 [23], 2020 [24], 2016 [25, 26], 2015 [27], and 2013 [28]. All studies were conducted in the USA in various states. All selected studies chose postmenopausal women between the ages of 50 and 75 years. The duration of studies varied between four [14–16, 18, 19, 23] to eight [25–27] weeks. The number of participants ranged from 12 [28] to 51 [24] individuals.

**Table 1:** Characteristics of included studies in the systematic review

Studies	Country	Study Design	Participant/ Baseline health status	Sample size		Trial Duration (Week)	Means Age		Means BMI		Intervention			Main outcomes	Exercise
				IG	CG		IG	CG	IG	CG	Type	Dose (g/day)	Control group		
Kang et al. 2024 [14]	USA	Parallel, R, DB, PC	28/ Hypertensive- overweight or obese	14	14	4	61 ± 7	64 ± 5	29.8 ± 4	30.2 ± 6	L-citrulline	10	Placebo (maltodextrin)	significant increase in FMD and reduced aortic systolic BP at rest and improved exercise BF, VC, TSI, and HHb	rhythmic handgrip exercise at 30% of MVC for 3 min with a metronome-controlled rate
Dillon et al. 2024 [16]	USA	Parallel, R, DB, PC	28/ Hypertensive-Overweight or obese	14	14	4	60	62	29.2	31.8	L-citrulline	10	Placebo (maltodextrin)	significant decrease in aortic SBP, PP, Pb no effect on resting BP and on aortic BP responses to IHG.	Metaboreflex / 2 min of IHG at 30% of their maximal voluntary contractions
Figueroa et al. 2023 [19]	USA	Parallel, R, DB, PC	44/ Hypertensive-Overweight or obese	13	17	4	58 ± 4	60 ± 5	29 ± 5	29.3 ± 3	L-citrulline	6	Placebo (Crystalline cellulose)	Significant changes (∆) in brachial SBP, brachial MAP, aortic SBP, and aortic MAP responses to the supplementation reduced ∆SBP and ∆MAP	-
Maharaj et al. 2022 (A) [12]	USA	Crossover, R, DB, PC	15/ Hypertensive-obese	4	11	Acute	65 ± 5	60 ± 8	30.9 ± 4	31.6 ± 5	L-citrulline	6	Placebo (maltodextrin)	Changes in brachial SBP and MAP, and aortic SBP and MAP from rest to EX + CPT were attenuated	calf-raise 10 repetition maximum
Maharaj et al. 2022 (B) [15]	USA	Parallel, R, DB, PC	28/ Hypertensive-obese or overweight	14	14	4	61 ± 6	64 ± 6	29.9 ± 4	30.9 ± 5	L-citrulline	10	Placebo (maltodextrin)	Baseline or peak brachial artery diameter and shear rate were not affected. significant increase in FMD No significant changes in aortic stiffness (cfPWV)	-
Kang et al. 2023 [18]	USA	Parallel, R, DB, PC	28/ Hypertensive-overweight or obese	14	14	4	62 ± 2	63 ± 1	29.6 ± 1	29.2 ± 2	L-citrulline	10	Placebo (maltodextrin)	significant increase in sfemFMD did not affect faPWV, but both groups had similar significant reductions in faPWV after the combination with SVLIRT	10 repetition maximum testing (leg press, leg extension, leg curl, and calf raise)
Ellis et al. 2021 [23]	USA	Crossover, R, DB, PC	21/ Blood pressure < 40/90 mm Hg -non-obese	9	12	4	60 ± 4	60 ± 4	25.05 ± 3	25.05 ± 3	Watermelon juice	1.63 ± 0.1	water, sucrose, non-nutritive watermelon flavoring, malic acid, pectin, cellulose, and food coloring	No significant result on vascular outcomes. did not appreciably affect BMI or percent body fat significant increase in fasting serum glucose HOMA-IR was in the same direction, it was not statistically significant	Blood pressure < 40/90 mm Hg -non-obese
Shanely et al. 2020 [24]	USA	Parallel, R	51/ Non-hypertensive-Overweight or obese	26	19	6	59.5 ± 1	60.1 ± 2	30.9 ± 1	30.3 ± 1	watermelon puree	2.28	Apple juice, sucrose-glucose-fructose beverage, or low-fat cookie	increased fasting plasma L-arginine, cis-lycopene, and trans-lycopene concentrations and decreased sVCAM-1 plasma concentration, fasting blood glucose, insulin, and HOMA-IR did not change	-
Wong et al. 2016 (A) [25]	USA	Parallel, R, PC	23/ Hypertensive-Obese	12	11	8	58 ± 1	58 ± 1	32.2 ± 1	32.9 ± 1	L-citrulline	6	Placebo (maltodextrin)	Significant Decrease in BP, LnLF/LnHF. a significant decrease in nLF and increase in nHF increased LnRMSSD but the change was not different compared to the control. no significant changes in LnTP and R-R intervals	-
Figueroa et al. 2015 [27]	USA	Parallel, R	41/ over- weight or obese with prehypertension or stage-1 hypertension	13	14	8	58 ± 1	58 ± 1	33.8 ± 1	35 ± 1	L-citrulline	6	Placebo (maltodextrin)	decrease in cfPWV compared with no significant changes in aortic stiffness, decreased faPW, baPWV Body weight, BMI, arm LM, and arm strength did not change increased leg LM and ALMI muscle strength increased	Metaboreflex- IHG was performed with the dominant arm at 30% of MVC for 2 min using a handgrip dynamometer
Wong et al. 2016 (B) [26]	USA	Parallel, R, PC	41/ overweight or obese – some non-hypertensive and some stage-1 hypertension or hypercholesterolemia	13	14	8	58 ± 3	58 ± 4	33.8 ± 4	35 ± 3	L-citrulline	6	Placebo (maltodextrin)	decreased brachial and aortic pressures as well as AP increased NOx levels. AIx and AIx@75 decreased in both groups	different exercises for the legs(static and dynamic squats )- duration of the exercise set (30–60 s), number of sets (1–5), and total duration of the training session (11–60 min)
Figueroa et al. 2013 [28]	USA	Crossover, R	12/ stage 1 hypertension-Obese	12	12	6	57	57	38.1	38.1	Lcitrulline/L-arginine	6	sucrose, glucose, and fructose	Significant decrease in aortic SBP, aortic DBP, aortic SBP, aortic SBP2, radial SBP1, and rSBP2. No significant changes in aortic AIx, Tr, radial AIx, and heart rate	-

*Abbreviations*: *IG *intervention group, *CG *control group, *DB *double-blinded, *R *randomized, *PC *placebo-controlled, *BMI *body mass index, *FMD *flow-mediated dilation, * BF *blood flow, *BP *blood pressure, *VC *vascular conductance, *TSI *tissue oxygen saturation index, *HHb *deoxygenated hemoglobin, *SBP *systolic blood pressure, *PP *pulse pressure, *Pb *pressure of backward, *IHG *isometric handgrip exercise, *MAP* mean arterial pressure, *cfPW *carotid-femoral pulse wave velocity, *SVLIRT *slow velocity low-intensity resistance training, *sfemFMD *superficial femoral artery flow-mediated dilation, *faPWV* femoral–ankle pulse wave velocity, *EX *exercise, *CPT *cold pressor test, * HOMA-IR *homeostasis model assessment-estimated insulin resistance, *sVCAM-1 *soluble vascular cell adhesion molecule-1, *nLF *sympathetic activity, *LnLF/LnHF* sympathovagal balance, *nHF *vagal tone, *RMSSD *root mean square of successive differences, *Ln* natural logarithm, *TP *total power, *LM *lean mass, *ALMI *appendicular lean mass index, *NOx *nitric oxide metabolites, *Aix *augmentation pressure index

### Blood pressure

Seven out of twelve studies examined the effect of citrulline on blood pressure. In one study by Dillion et al. [16], citrulline significantly reduced pulse pressure (PP) (− 5 ± 2 v. 0 ± 1 mmHg, *P* = 0·03), systolic blood pressure (SBP) (− 9 ± 2 v. −1 ± 1 mmHg, *P* = 0·006), backward pressure (Pb) (− 3 ± 1 v. 0 ± 1 mmHg, *P* = 0·02), and forward pressure (Pf) (− 6 ± 2 v. −1 ± 1 mmHg, *P* = 0·01), although resting BP remained unchanged. A similar reduction in SBP was reported in other studies [12, 19, 26, 28]. Maharaj et al. [12] also found significant decreases in brachial and aortic mean arterial pressure (MAP) from rest to exercise. Another study [14] reported lower resting aortic systolic BP (∆3 ± 5 vs. ∆−4 ± 6 mmHg *p* < 0.05), but no significant changes in brachial BP during exercise (all *p* > 0.05). This study also suggested that citrulline significantly reduced brachial SBP, MAP, and aortic MAP compared to placebo (all *p* < 0.05). One study indicated that citrulline alone or combined with whole-body vibration training increased nitric oxide metabolites (NOx) and reduced SBP, DBP, aortic PP, and brachial PP(*p* < 0.05) [26]. Wong et al. [25] observed a significant group-by-time interaction for LnLf/LnHF(*p* < 0.05) that decreased. also, LnRMSSD increased following citrulline supplementation compared to the control group. In contrast, Ellis et al. [23] reported no significant changes in any vascular outcomes. Overall, citrulline modestly reduced systolic-related BP indices, but effects on resting BP and exercise responses were inconsistent.

### Arterial stiffness

Five out of twelve studies evaluated the effect of citrulline on arterial stiffness, with mixed and sometimes conflicting findings. Figueroa et al. [27] suggested that citrulline supplementation combined with whole-body vibration training (WBVT) decreased carotid-femoral pulse wave velocity (cfPWV), whereas citrulline supplementation alone did not significantly alter arterial stiffness during combined metaboreflex activation and cold exposure, despite reductions in Femoral-ankle PWV (faPWV) and brachial-ankle PWV (baPWV) (*P* < 0·05). Maharaj et al. [15] also reported a non-significant decrease in cfPWV after supplementation. Another trial [28] observed a significant reduction in baPWV (-1.2 [0.3] m/s, *P* < 0.001) after watermelon supplementation, which correlated with the changes in aortic SBP P (*r* = 0.64, P 0.01), SBP2 (*r* = 0.64, *P* < 0.01), and rSBP2(*r* = 0.57, *P* < 0.01). Kang et al. [18] observed no significant effect of citrulline on faPWV, although reductions occurred when combined with slow velocity low-intensity resistance training (SVLIRT). Another parameter for assessing arterial stiffness is Augmentation Index (AIx); two studies assessed this parameter [26, 28]. One study suggested that citrulline supplementation in combination with whole-body vibration training decreased AIx and AIx@75 (− 10.5% ± 8.8%, *P* < 0.05) [26], while the other study observed no significant changes in aortic AIx, reflection time (Tr), and radial AIx [28]. Overall, citrulline showed small improvements in arterial stiffness, mainly with exercise-based interventions, while findings for citrulline alone remained inconsistent.

### Endothelial function

Four out of twelve studies investigated the effect of citrulline on endothelial function. Two trials [14, 18] demonstrated a significant increase in flow-mediated dilation (FMD) (∆1.6 ± 0.7%, *p* < 0.05) and sfemFMD (∆1.8 ± 0.3%, *p* < 0.05) following citrulline versus placebo. Figueroa et al. [19] found that citrulline combined with glutathione (GSH) further improved FMD (*p* < 0.05). Overall, citrulline supplementation improved endothelial function in most studies, particularly in FMD measures.

### Muscle function

Two out of twelve studies investigated the effect of citrulline on Muscle function. Figueroa et al. [27] found no significant effects of citrulline on arm lean mass (LM), arm strength, leg strength, leg LM, and Appendicular Lean Mass Index (ALMI); however, citrulline combined with WBVT significantly increased leg LM (6.0 ± 2.2%, *P* < 0.05) and ALMI (4.8 ± 1.8%, *P* < 0.05). Kang et al. [18] reported similar improvements in leg LM, leg curl, and leg strength (*p* < 0.05). Overall, citrulline alone had minimal effects on muscle outcomes, but showed benefits when combined with training.

### Metabolic parameters

Six out of twelve studies investigated the effect of citrulline on Metabolic parameters. Ellis et al. [23] and Figueroa et al. [19] reported no significant effect on FMD, whereas other trials observed significant changes in body weight, BMI, glucose, insulin, and low-density lipoprotein (LDL) [18, 23–25, 27, 28] after citrulline supplementation. One study showed that fasting serum glucose increased (*p* = 0.001) after four weeks of watermelon juice; however, the peak did not meet the World Health Organization’s threshold for impaired fasting glucose (110 mg/dL) [23]. Conversely, fasting blood glucose, fasting blood insulin, and Homeostatic Model Assessment for Insulin Resistance (HOMA-IR) did not change significantly after six weeks of watermelon puree supplementation [24]. Overall, metabolic effects were variable, with some improvements reported but inconsistent across studies.

### Quality assessment

The risk of bias assessment, conducted using the Cochrane Risk of Bias Tool for Randomized Trials (RoB 2), revealed that the majority of included studies (7 out of 12) demonstrated a low risk of bias across all domains [12, 14–16, 18, 19, 23]. Three studies [24, 26, 28] were rated as having “some concerns”, primarily due to unclear reporting of protocol deviations or outcome measurement methods. Two studies [25, 27] were classified as high risk due to significant issues such as missing data and inadequate blinding (Table 2). Overall, the predominance of low-risk studies supports the reliability of the main findings; however, the presence of studies with some concerns and high risk underscores the need for cautious interpretation, particularly for outcomes with conflicting results. Consequently, findings from studies with low risk of bias should be weighted more heavily, especially for blood pressure outcomes, where most studies were low risk (Supplementary Table 3).

**Table 2:** Risk of bias assessment

Study	Bias arising from the randomization process	Bias in selection of the reported result	Bias due to deviations from intended interventions	Bias in measurement of the outcome	Bias due to missing outcome data	Overall risk of bias
Kang et al. 2024 [14]	L	L	L	L	L	Low
Dillon et al. 2024 [16]	L	L	L	L	L	Low
Figueroa et al. 2023 [19]	L	L	L	L	L	Low
Maharaj et al. 2022 (A) [12]	L	L	L	L	L	Low
Maharaj et al. 2022 (B) [15]	L	L	L	L	U	Low
Kang et al. 2023 [18]	L	L	L	L	L	Low
Ellis et al. 2021 [23]	L	L	L	L	L	Low
Shanely et al. 2020 [24]	L	L	U	U	L	Some concerns
Wong et al. 2016 (A) [25]	L	H	U	U	L	High
Figueroa et al. 2015 [27]	L	L	U	U	H	High
Wong et al. 2016 (B) [26]	L	L	U	U	L	Some concerns
Figueroa et al. 2013 [28]	L	L	U	U	L	Some concerns

*L* low risk of bias, *H* high risk of bias, *U* unclear risk of bias

Overall Low Risk < 2 unclear risk of bias and no high risk of bias;

Overall Some concerns = 2 unclear risk of bias and no high risk of bias

Overall High Risk > 2 unclear risk of bias or more than one high risk of bias

### Safety

None of the included studies based on the dosage of the supplements reported any adverse effects.

## Discussion

This review included 12 RCTs. Most studies showed that systolic blood pressure and mean arterial pressure were reduced with citrulline supplementation. Two studies showed improvement in flow-mediated dilation. Two studies examined muscle performance outcomes. They showed that citrulline supplementation improved outcomes when combined with resistance exercise. No effects were seen on metabolic parameters such as weight, insulin, glucose, and lipid profiles.

Several studies found that citrulline supplementation (10 g/day for 4 weeks) significantly reduced aortic diastolic blood pressure and mean arterial pressure, and improved endothelial function in hypertensive postmenopausal women [14, 15]. Another study showed that combining citrulline with glutathione attenuated blood pressure reactivity to stress, but citrulline alone did not significantly affect resting blood pressure in healthy postmenopausal women [19] (Table 3).

**Table 3:** Directionality and statistical significance of the effects of citrulline supplementation across included randomized trials

Metabolic Parameters	Muscle Function	Arterial Stiffness (PWV / AIx)	Endothelial Function (FMD)	Blood Pressure	Study
→	→	→	↑ (sig)	↓ (sig)	Kang et al., 2024 [14]
–	–	→	→	↓ (sig DP / PP)	Dillon et al., 2024 [16]
→	–	→	↑ (sig only with GSH)	↓ (sig reactivity)	Figueroa et al., 2023 [19]
–	–	→	→	↓ (sig reactivity)	Maharaj et al., 2022 (A) [12]
–	–	→	↑ (sig)	↓ (sig)	Maharaj et al., 2022 (B) [15]
→	↑ (with training)	→ (alone) / ↓ (sig with training)	↑ (sig)	→	Kang et al., 2023 [18]
↑ (fasting glucose)	–	→	→	→	Ellis et al., 2021 [23]
→	–	–	–	→	Shanely et al., 2020 [24]
–	–	–	–	↓ (sig)	Wong et al., 2016 (A) [25]
→	↑ (leg LM with WBVT)	↓ (cfPWV, baPWV)	–	↓ (sig)	Figueroa et al., 2015 [27]
–	–	↓ AIx (both groups)	–	↓ (sig)	Wong et al., 2016 (B) [26]
–	–	→	→	↓ (sig)	Figueroa et al., 2013 [28]

A systematic review and meta-analysis concluded that citrulline supplementation had no significant effect on brachial or aortic blood pressure overall, with body weight status identified as a source of heterogeneity [29].

In hypertensive postmenopausal women, citrulline supplementation showed a trend toward reduced aortic stiffness, but the change was not statistically significant [15]. Combined supplementation with glutathione did not affect aortic stiffness [19].

Reasons for different results are supplementation Protocols: Dose (1.5–10 g/day), duration (4–8 weeks), and combination with other interventions (e.g., glutathione, HIIT, nitrate) influenced outcomes [15, 19, 30]. Endpoints Measured: Some studies focused on resting blood pressure, others on stress-induced responses or exercise conditions, which may yield different results [19]. Baseline Vascular Health: Greater benefits were observed in individuals with endothelial dysfunction or higher baseline arterial stiffness [14, 15]. Meta-Analysis Findings: Heterogeneity in study populations, particularly body weight, contributed to inconsistent results across studies [29].

A comparative analysis of study characteristics reveals patterns that may explain the divergent findings. Studies reporting significant vascular improvements, such as reduced blood pressure or enhanced endothelial function [14, 15, 19], typically administered higher doses of pure L-citrulline (6–10 g/day) to participants with confirmed hypertension or endothelial dysfunction. In contrast, trials showing null effects, such as Ellis et al. [23], often utilized lower doses of citrulline derived from watermelon (≈ 1.6 g/day) in normotensive, healthier cohorts. Furthermore, the method of outcome assessment appears influential. Studies measuring blood pressure reactivity to physiological stress (e.g., exercise, cold pressor test) [12, 16, 19] were more consistently positive than those assessing only resting hemodynamics [23]. This suggests that citrulline’s vasodilatory effects may be more readily unmasked during cardiovascular challenge, particularly in individuals with pre-existing vascular impairment. The source (pure supplement vs. food matrix), dose, participant baseline health, and the dynamic nature of the outcome measurement collectively form a framework for interpreting the efficacy of citrulline supplementation in this population.

Citrulline supplementation alone in postmenopausal women, especially those with hypertension, may improve endothelial function and muscle blood flow during exercise, but its effects on muscle mass and strength are limited unless combined with resistance training. Studies show that citrulline supplementation increases leg muscle oxygenation, arterial vasodilation, and blood flow during exercise, which may support muscle function indirectly by enhancing nutrient and oxygen delivery to muscles [14, 17].

When combined with resistance training (such as whole-body vibration or low-intensity resistance training), citrulline supplementation leads to greater improvements in leg lean mass and muscle strength compared to either intervention alone [18, 27, 31]. The combination also enhances endothelial function and reduces arterial stiffness, which are important for muscle health in this population [18, 27, 31]. This pattern aligns with findings from other supplement-exercise combinations across diverse nutritional agents, including saffron with circuit resistance training [32], spinach-derived thylakoids with high-intensity training [33], and spirulina with high-intensity interval training [34], suggesting that nutritional support can potentiate exercise-induced benefits across different supplement classes, exercise modalities, and health domains.

Menopausal women experience reduced estrogen, leading to lower nitric oxide synthesis and endothelial dysfunction, which impairs muscle blood flow and function. Citrulline supplementation helps by increasing arginine availability and nitric oxide production, thus improving vascular function and, when paired with exercise, muscle mass and strength [17, 18, 27, 31]. Multiple studies demonstrate that citrulline supplementation significantly raises serum L-arginine levels and improves FMD, a marker of endothelial-dependent vasodilation, in hypertensive and healthy postmenopausal women [14, 15, 17, 18, 35–37]. Citrulline, especially when combined with antioxidants, increases the NO/peroxynitrite ratio, indicating enhanced NO bioavailability and reduced oxidative stress in endothelial cells [19, 37, 38]. Citrulline inhibits arginase activity, which otherwise competes with nitric oxide synthase (eNOS) for L-arginine, further supporting NO production under conditions of oxidative stress and endothelial dysfunction [6, 39]. Combining citrulline with glutathione or other antioxidants further improves FMD and blood pressure reactivity, suggesting that addressing oxidative stress enhances the efficacy of citrulline in restoring NO-mediated dilation [19, 37]. Discrepancies in FMD findings may relate to methodological differences in ultrasound measurement, baseline endothelial function, supplementation dose (6–10 g/day in positive studies vs. watermelon-derived citrulline in negative studies), and participant characteristics (hypertensive vs. normotensive).

The impact of citrulline supplementation on vascular and metabolic outcomes in postmenopausal women is strongly influenced by baseline health status. Benefits are most pronounced in women with hypertension, prehypertension, obesity, or endothelial dysfunction, while effects in healthy or normotensive women are limited.

Overall, citrulline supplementation offers modest but meaningful benefits for blood pressure, endothelial function, and muscle health, especially in older or at-risk populations. These effects support its use as a non-pharmaceutical strategy to improve cardiovascular and functional health at both individual and public health levels.

Significant heterogeneity across included studies limits definitive conclusions and warrants careful interpretation. The wide dose range (1.5–10 g/day) may explain variability in outcomes, with studies using higher doses (≥ 6 g/day) more consistently reporting improvements in blood pressure and endothelial function compared to those using lower doses or watermelon-derived citrulline. This suggests a potential dose-dependent effect, though optimal dosing remains undefined. Citrulline source also appears influential: pure L-citrulline supplements typically provided higher daily doses (6–10 g) and showed more consistent vascular benefits, whereas watermelon products delivered lower citrulline equivalents (1.5–2.3 g/day) and yielded mixed results, possibly due to additional bioactive compounds (e.g., lycopene, vitamin C) exerting independent effects. Intervention duration (4–8 weeks) showed no clear relationship with outcome magnitude, suggesting that even short-term supplementation may elicit physiological changes in this population. Importantly, concomitant interventions substantially modulated results: citrulline alone showed modest vascular benefits, but combining it with resistance training produced additive improvements in muscle mass and strength, while pairing with glutathione enhanced endothelial function and blood pressure reactivity. These subgroup patterns highlight that citrulline’s efficacy is context-dependent and influenced by dosage, formulation, and complementary interventions.

### Mechanism of action of citrulline

#### Nitric oxide pathway and endothelial function

Citrulline acts primarily by increasing the availability of L-arginine, which is the substrate for endothelial eNOS. This leads to enhanced production of NO, a potent vasodilator that improves endothelial function and vascular tone. Unlike L-arginine, citrulline is not extensively metabolized in the gut or liver, making it more effective at raising plasma L-arginine and NO levels [6, 15, 40, 41]. Improved NO bioavailability results in better FMD, indicating enhanced endothelial function [14, 15, 19, 42].

L-citrulline is more effective than L-arginine at increasing plasma arginine levels and NO synthesis because it bypasses first-pass metabolism in the gut and liver, leading to greater systemic availability [14, 43]. Oral L-arginine is largely metabolized before reaching systemic circulation, limiting its effectiveness [14, 43].

#### Reduction of blood pressure

By increasing NO production, citrulline supplementation leads to vasodilation, which lowers both resting and stress-induced blood pressure, particularly in individuals with prehypertension or hypertension [6, 14, 15, 40, 41]. Studies show significant reductions in aortic and brachial blood pressures after citrulline supplementation, attributed to improved endothelial function and increased L-arginine/NO pathway activity [14, 15, 19, 41].

Both L-citrulline and L-arginine supplements can lower blood pressure, but L-citrulline may have a more consistent effect, especially in populations with elevated blood pressure [15, 44, 45]. L-citrulline supplementation has shown improvements in endothelial function and arterial stiffness, while L-arginine’s effects are less reliable due to the “L-arginine paradox”, where increased plasma arginine does not always translate to increased NO production [15, 43–45].

#### Decrease in vascular stiffness

Citrulline supplementation has been shown to reduce arterial stiffness, as measured by PWV and baPWV. This effect is linked to increased NO production, which relaxes vascular smooth muscle and improves arterial compliance [15, 42].

#### Enhancement of muscle function

Citrulline improves muscle function by increasing blood flow and oxygen delivery to skeletal muscles during exercise. This is achieved through NO-mediated vasodilation, which enhances muscle oxygenation and nutrient delivery, leading to better exercise performance and muscle health [6, 14, 40, 46]. NO increases blood flow, nutrient delivery, and cellular signaling, which together can enhance muscle protein synthesis, mitochondrial biogenesis, and fatigue resistance. Increased muscle tissue oxygen saturation and reduced deoxygenated hemoglobin during exercise have been observed with citrulline supplementation [14, 40].

L-citrulline supplementation, particularly at doses of 2.4–6 g/day for 7–16 days, has been shown to improve exercise performance, reduce perceived exertion, and enhance muscle oxygenation more effectively than L-arginine [47–49]. Some studies report that combining L-citrulline and L-arginine may have synergistic effects, further improving NO levels and exercise outcomes [50, 51]. However, acute supplementation with either amino acid does not consistently improve performance in all exercise modalities [52].

### Limitations

While this systematic review provides valuable insights into the effects of citrulline supplementation in postmenopausal women, several limitations should be acknowledged. The relatively small number of RCTs available for each outcome measure (e.g., blood pressure, arterial stiffness, muscle function) precluded a quantitative meta-analysis, limiting the ability to draw robust statistical conclusions. The search was limited to three databases, potentially missing relevant studies from other sources. Significant heterogeneity existed across studies in terms of citrulline dosage (1.5–10 g/day), intervention duration (4–8 weeks), and participant characteristics (e.g., baseline health status, age range), which may influence the generalizability of the findings. Additionally, watermelon-derived citrulline provides additional bioactive compounds (lycopene, vitamin C, antioxidants) that may exert independent effects, limiting direct comparison between watermelon-derived and pure citrulline supplements. In one study, the risk of bias for selective reporting appears to be high due to reporting outcomes that increase the overall conclusion for the positive effect of citrulline on sympathovagal balance in obese postmenopausal women [25], and this has negatively affected the quality of the study and has also affected the certainty of the overall conclusion of our study. Publication bias was not formally assessed due to the narrative synthesis approach and small number of studies per outcome. All 12 studies were conducted in the United States, which significantly limits generalizability. Additionally, several studies evaluated citrulline in combination with other interventions (e.g., resistance training, glutathione), making it difficult to isolate the independent effects of citrulline supplementation. Furthermore, the predominance of short-term studies (≤ 8 weeks) means long-term safety data are lacking. Future research should prioritize larger, long-term RCTs with standardized protocols to strengthen the evidence base.

### Clinical implication

Citrulline supplementation may be considered for postmenopausal women with hypertension or endothelial dysfunction, where doses of 6–10 g/day for 4–8 weeks have demonstrated modest blood pressure reductions and improved vascular function. For muscle health, it appears most beneficial when combined with resistance training. However, routine clinical use is not yet supported due to gaps in long-term safety data, heterogeneous optimal dosing, and insufficient evidence for metabolic benefits. Future research should prioritize standardized, long-term trials before therapeutic recommendations can be established.

## Conclusion

Citrulline supplementation may help to reduce blood pressure and vascular stiffness in postmenopausal women, particularly when combined with other interventions and in those with hypertension or endothelial dysfunction. In some studies, citrulline supplementation improved muscle function. These effects are caused by boosting L-arginine and NO production, leading to enhanced vasodilation, improved endothelial function, and increased muscle blood flow and oxygenation. However, due to significant heterogeneity, the small number of studies and sample size per outcome, no definitive conclusions can be drawn, and more RCTs are needed in this regard.

## Supplementary Information

Supplementary Material 1.

Supplementary Material 2.

Supplementary Material 3.

Supplementary Material 4.

## Data Availability

All data generated or analyzed during this study are included in this published article.
