# Supplementary material for: Citrulline supplementation in postmenopausal women: a systematic review of vascular, muscular, and metabolic effects
Source: BMC Womens Health. 2026 Jan 26;26:116. doi: 10.1186/s12905-026-04277-6 (PMC12918617; doi:10.1186/s12905-026-04277-6)
Supplement: Supplementary file 1 — Supplementary Material 1. [file 12905_2026_4277_MOESM1_ESM.docx]

**Supplementary Table 1**. Complete search strategies

| PubMed | ("citrulline"[tiab] OR "L-citrulline"[tiab] OR "watermelon"[tiab] OR "Citrullus vulgaris"[tiab])  AND  ("postmenopausal"[tiab] OR "post-menopausal"[tiab] OR "menopausal"[tiab] OR "menopause"[tiab] OR "postmenopause"[tiab] OR "post-menopause"[tiab])  AND  ("randomized controlled trial"[pt] OR "randomised controlled trial"[pt] OR "clinical trial"[pt] OR "randomized"[tiab] OR "randomised"[tiab] OR "RCT"[tiab] OR "trial"[tiab] OR "clinical study"[tiab]) |
| --- | --- |
| Scopus | TITLE-ABS-KEY(("citrulline" OR "L-citrulline" OR "watermelon" OR "Citrullus vulgaris")  AND  ("postmenopausal" OR "post-menopausal" OR "menopausal" OR "menopause" OR "postmenopause" OR "post-menopause")  AND  ("randomized controlled trial" OR "randomised controlled trial" OR "clinical trial" OR "randomized" OR "randomised" OR "RCT" OR "trial" OR "clinical study")) |
| Web of Science | TS=(("citrulline" OR "L-citrulline" OR "watermelon" OR "Citrullus vulgaris")  AND  ("postmenopausal" OR "post-menopausal" OR "menopausal" OR "menopause" OR "postmenopause" OR "post-menopause")  AND  ("randomized controlled trial" OR "randomised controlled trial" OR "clinical trial" OR "randomized" OR "randomised" OR "RCT" OR "trial" OR "clinical study")) |
