# Supplementary material for: Citrulline supplementation in postmenopausal women: a systematic review of vascular, muscular, and metabolic effects
Source: BMC Womens Health. 2026 Jan 26;26:116. doi: 10.1186/s12905-026-04277-6 (PMC12918617; doi:10.1186/s12905-026-04277-6)
Supplement: Supplementary file 3 — Supplementary Material 3. [file 12905_2026_4277_MOESM3_ESM.docx]

| **Supplementary Table 2.** A summary of excluded articles after full-text review | |
| --- | --- |
| **Author, Year (Ref.)** | **Reason** |
| Theodorou, 2021 (1) | Due to Not interested population |
| Caballero-García, 2021 (2) | Due to Not interested outcome |
| Aguayo, 2021 (3) | Review study |
| Dillon, 2024 (4) | Abstract |
| Figueroa, 2016 (5) | Due to Not interested population |
| Agarwal, 2017 (6) | Animal study |
| Tryfonos, 2023 (7) | Due to Not interested population |
| Tovar-Villegas, 2024 (8) | Due to Not interested population |

1. Theodorou AA, Zinelis PT, Malliou VJ, Chatzinikolaou PN, Margaritelis NV, Mandalidis D, et al. Acute L-Citrulline Supplementation Increases Nitric Oxide Bioavailability but Not Inspiratory Muscle Oxygenation and Respiratory Performance. Nutrients. 2021;13(10).

2. Caballero-García A, Pascual-Fernández J, Noriega-González DC, Bello HJ, Pons-Biescas A, Roche E, Córdova-Martínez A. L-Citrulline Supplementation and Exercise in the Management of Sarcopenia. Nutrients [Internet]. 2021; 13(9).

3. Aguayo E, Martínez-Sánchez A, Fernández-Lobato B, Alacid F. L-Citrulline: A Non-Essential Amino Acid with Important Roles in Human Health. Applied Sciences [Internet]. 2021; 11(7).

4. Dillon K, Kang YJ, Martinez M, Figueroa A. L-Citrulline Supplementation Improves Leg Endothelial Function and Blood Flow Responses to Plantarflexion Exercise in Hypertensive Postmenopausal Women. Physiology. 2024;39.

5. Figueroa A, Alvarez-Alvarado S, Jaime SJ, Kalfon R. L-Citrulline supplementation attenuates blood pressure, wave reflection and arterial stiffness responses to metaboreflex and cold stress in overweight men. British Journal of Nutrition. 2016;116(2):279-85.

6. Agarwal U, Didelija IC, Yuan Y, Wang X, Marini JC. Supplemental Citrulline Is More Efficient Than Arginine in Increasing Systemic Arginine Availability in Mice. J Nutr. 2017;147(4):596-602.

7. Tryfonos A, Christodoulou F, Pamboris GM, Christodoulides S, Theodorou AA. Short-Term L-Citrulline Supplementation Does Not Affect Blood Pressure, Pulse Wave Reflection, or Arterial Stiffness at Rest and during Isometric Exercise in Older Males. Sports. 2023;11(9).

8. Tovar-Villegas VI, Kang YJ, Ibarra-Reynoso LDR, Olvera-Juárez M, Gomez-Ojeda A, Bosquez-Mendoza VM, et al. Oral L-Citrulline Supplementation Improves Fatty Liver and Dyslipidemia in Adolescents with Abdominal Obesity: A Parallel, Double-Blind, Randomized Clinical Trial. Gastroenterology Insights. 2024;15(2):354-65.
