# Supplementary material for: Citrulline supplementation in postmenopausal women: a systematic review of vascular, muscular, and metabolic effects
Source: BMC Womens Health. 2026 Jan 26;26:116. doi: 10.1186/s12905-026-04277-6 (PMC12918617; doi:10.1186/s12905-026-04277-6)
Supplement: Supplementary file 4 — Supplementary Material 4. [file 12905_2026_4277_MOESM4_ESM.docx]

**Supplementary Table 3.** Detailed risk of bias assessment for each included study using the Cochrane RoB 2 tool.

| **Study** | **Bias arising from the randomization process** | **Bias in selection of the reported result** | **Bias due to deviations from intended interventions** | **Bias in measurement of the outcome** | **Bias due to missing outcome data** | **Overall risk of bias** |
| --- | --- | --- | --- | --- | --- | --- |
| Kang et al. 2024 (1) | L | L | L | L | L | Low |
|  | Participants were randomized using an investigator-independent, computer-based sequence stratified by age and systolic blood pressure, with comparable baseline characteristics between groups. | The study was prospectively registered on ClinicalTrials.gov and reported outcomes were consistent with the prespecified analyses. | Although blinding to the WBVT intervention was not feasible, there was no evidence that awareness of group assignment led to deviations from the intended interventions | Outcomes were objectively measured (ultrasound, NIRS, BP devices) | All randomized participants completed the study, compliance was high (>93%), and no outcome data were missing. |  |
| Dillon et al. 2024 (2) | L | L | L | L | L | Low |
|  | Participants were randomly assigned to citrulline or placebo using a block randomization scheme stratified by age and brachial systolic blood pressure, and baseline characteristics were similar between groups. | The study was prospectively registered (ClinicalTrials.gov), and reported outcomes and analyses were consistent with the prespecified objectives, with no evidence of selective reporting. | The study was double-blind and placebo-controlled, with identical capsules and high compliance (>90%), and no evidence of deviations from the intended intervention that could affect outcomes. | Aortic blood pressure and pressure wave variables were assessed using validated applanation tonometry methods, and outcome measurements were objective and unlikely to be influenced by knowledge of group assignment. | Most participants completed the study, and missing outcome data were minimal and due to technical issues, which are unlikely to be related to the true intervention effects. |  |
| Figueroa et al. 2023 (3) | L | L | L | L | L | Low |
|  | Participants were randomly assigned using a computer-generated block randomization scheme stratified by age and systolic blood pressure, and baseline characteristics were largely comparable across groups. | The study was prospectively registered on ClinicalTrials.gov, and reported outcomes and analyses were consistent with the prespecified endpoints, with no evidence of selective reporting. | The study was double-blind and placebo-controlled, with indistinguishable capsules and high adherence (>94%), and no protocol deviations or contamination between groups were reported. | Outcomes were assessed using objective, validated methods (e.g., ultrasound-based FMD, applanation tonometry), and outcome assessors were blinded to group allocation. | Attrition was low, reasons for missing data were reported, and outcome data were available for the majority of randomized participants, making bias from missing data unlikely. |  |
| Maharaj et al. 2022 (A) (4) | L | L | L | L | L | Low |
|  | This study used a randomized, double-blind, placebo-controlled, crossover design. Participants were randomly assigned to the order of L-citrulline or placebo conditions, with a washout period of at least three days between conditions. | The study was prospectively registered, and the reported outcomes and statistical analyses were consistent with the prespecified objectives and methods, with no evidence of selective outcome reporting. | Both participants and investigators were blinded to treatment allocation. L-citrulline and placebo supplements were identical in appearance and administered under controlled conditions | Aortic systolic blood pressure was assessed using validated, noninvasive tonometry and transfer-function methods, which are widely accepted for central blood pressure assessment | All participants completed both intervention conditions, and outcome data were available for the primary outcome |  |
| Maharaj et al. 2022 (B) (5) | L | L | L | L | U | Low |
|  | Participants were randomized (stratified by age and SBP) to L-CIT or placebo. Baseline characteristics (age, BMI, BP) were well-balanced between groups. No evidence of deviations from intended random allocation | Pre-specified outcomes were reported as planned, with study registration on ClinicalTrials.gov | Study was double-blind, with participants and study personnel unaware of group assignment.  High compliance reported. No major protocol deviations affecting outcome measurement reported. | Outcomes were objective (FMD, cfPWV, BP, serum L-ARG) and measured using standardized instruments. | Three participants dropped out, reasons not fully explained, and no sensitivity analysis was reported. |  |
| Kang et al. 2023 (6) | L | L | L | L | L | Low |
|  | Randomization was performed using a computer-generated sequence by an independent researcher, with balanced baseline characteristics across groups. | The study was prospectively registered, and the reported outcomes aligned with the pre-specified protocol, with no evidence of selective reporting.ch | High compliance rates (>90%) and no evidence of protocol deviations that impacted outcomes; adherence to supplementation and training protocols was well-maintained. | Outcomes were assessed using objective, standardized, and validated instruments applied equally across groups, and assessor awareness of intervention assignment was unlikely to influence outcome measurement. | All participants completed the study with minimal attrition, and missing data were negligible and balanced across groups. |  |
| Ellis et al. 2021 (7) | L | L | L | L | L | Low |
|  | Participants were randomized using a computer-generated blocked randomization scheme, with allocation concealed via a closed-envelope method, and baseline characteristics were comparable between intervention sequences. | The trial was prospectively registered (ClinicalTrials.gov: NCT03626168), and reported outcomes and analyses were consistent with the prespecified protocol. | The study was double-blind and placebo-controlled with indistinguishable beverages, high adherence was monitored, and no deviations from the intended interventions were reported. | Vascular outcomes were assessed using validated, objective methods (PWV, ABPM, FMD), and outcome assessors were blinded to treatment allocation. | Attrition was low, reasons for withdrawal were reported, and an intention-to-treat analysis was applied, making bias due to missing outcome data unlikely. |  |
| Shanely et al. 2020 (8) | L | L | U | U | L | Some concerns |
|  | The study is described as randomized, and baseline characteristics were similar between groups, suggesting that the randomization process was adequate. | The reported outcomes were consistent with the study objectives, and there was no clear evidence of selective outcome reporting. | The study was not blinded, and participants were aware of their group assignment; however, no clear evidence of deviations from the intended intervention that could have influenced the outcome was reported. | Although the outcome was measured using a laboratory assay, it was not clearly stated whether outcome assessors were blinded to group allocation. | Outcome data were available for most participants, and the amount of missing data was small and unlikely to be related to the outcome. |  |
| Wong et al. 2016 (A) (9) | L | H | U | U | L | High |
|  | Participants were randomly assigned using a computer-generated sequence stratified by BMI, and baseline characteristics were comparable between groups. | Although the trial was registered, the study did not clearly prespecify HRV outcomes or statistical analysis plans, raising concerns about selective reporting of significant results. | Although participants were randomized, the study does not clearly state that it was double-blinded, and awareness of group assignment by participants or personnel cannot be ruled out. | Heart rate variability outcomes were objectively measured; however, it was not explicitly stated whether outcome assessors were blinded to group allocation. | Outcome data were available for nearly all participants, compliance was very high (98%), and no differential attrition between groups was reported. |  |
| Figueroa et al. 2015 (10) | L | L | U | U | H | High |
|  | Participants were randomly assigned to intervention groups with stratification by BMI and systolic blood pressure, and baseline characteristics were comparable across groups. | The study was prospectively registered, and reported outcomes and analyses were consistent with the prespecified objectives and methods, with no evidence of selective reporting. | Although the study included placebo control for supplementation, blinding of participants and personnel was not clearly described, particularly for the exercise intervention, which could not be blinded. | Outcomes were measured using validated and objective techniques; however, it was not explicitly stated whether outcome assessors were blinded to group allocation. | Several participants were excluded from the final analysis after randomization, and no intention-to-treat analysis was reported, raising concerns about the impact of missing data on the results. |  |
| Wong et al. 2016 (B) (11) | L | L | U | U | L | Some concerns |
|  | Participants were randomized using a computer-generated sequence with BMI stratification, and baseline characteristics were balanced across groups. | The study was prospectively registered and reported outcomes were consistent with the prespecified protocol. | Blinding to the WBVT intervention was not feasible and an intention-to-treat analysis was not explicitly reported. | Outcomes were objectively measured, but assessor blinding was not clearly reported. | Attrition was low and balanced between groups, with missing data unrelated to the intervention or outcomes. |  |
| Figueroa et al. 2013 (12) | L | L | U | U | L | Some concerns |
|  | Participants were randomly assigned in a randomized cross-over design, and baseline characteristics before each intervention period were comparable, indicating an adequate randomization process. | Reported outcomes and analyses were consistent with the stated study objectives, and no evidence of selective outcome reporting was identified. | Although the study used placebo control and a cross-over design, blinding of participants and study personnel was not clearly described, and deviations from intended interventions cannot be fully ruled out. | Arterial stiffness and wave reflection outcomes were measured using validated and objective techniques; however, it was not explicitly stated whether outcome assessors were blinded to the intervention sequence. | All participants completed both intervention periods, compliance was high (>97%), and outcome data were available for nearly all randomized participants. |  |

L; low risk of bias; H, high risk of bias; U, unclear risk of bias

Overall Low Risk < 2 unclear risk of bias and no high risk of bias;

Overall Some concerns = 2 unclear risk of bias and no high risk of bias

Overall High Risk > 2 unclear risk of bias or more than one high risk of bias

1. Kang Y, Dillon KN, Martinez MA, Maharaj A, Fischer SM, Figueroa A. L-Citrulline Supplementation Improves Arterial Blood Flow and Muscle Oxygenation during Handgrip Exercise in Hypertensive Postmenopausal Women. Nutrients. 2024;16(12).

2. Dillon KN, Kang Y, Maharaj A, Martinez MA, Fischer SM, Figueroa A. L-Citrulline supplementation attenuates aortic pressure and pressure waves during metaboreflex activation in postmenopausal women. Br J Nutr. 2024;131(3):474-81.

3. Figueroa A, Maharaj A, Kang Y, Dillon KN, Martinez MA, Morita M, et al. Combined Citrulline and Glutathione Supplementation Improves Endothelial Function and Blood Pressure Reactivity in Postmenopausal Women. Nutrients. 2023;15(7).

4. Maharaj A, Fischer SM, Dillon KN, Kang YJ, Martinez MA, Figueroa A. Acute Citrulline Blunts Aortic Systolic Pressure during Exercise and Sympathoactivation in Hypertensive Postmenopausal Women. Medicine & Science in Sports & Exercise. 2022;54(5):761-8.

5. Maharaj A, Fischer SM, Dillon KN, Kang YJ, Martinez MA, Figueroa A. Effects of L-Citrulline Supplementation on Endothelial Function and Blood Pressure in Hypertensive Postmenopausal Women. Nutrients. 2022;14(20).

6. Kang Y, Dillon KN, Martinez MA, Maharaj A, Fischer SM, Figueroa A. Combined L-Citrulline Supplementation and Slow Velocity Low-Intensity Resistance Training Improves Leg Endothelial Function, Lean Mass, and Strength in Hypertensive Postmenopausal Women. Nutrients. 2023;15(1).

7. Ellis AC, Mehta T, Nagabooshanam VA, Dudenbostel T, Locher JL, Crowe-White KM. Daily 100% watermelon juice consumption and vascular function among postmenopausal women: A randomized controlled trial. Nutr Metab Cardiovasc Dis. 2021;31(10):2959-68.

8. Shanely RA, Zwetsloot JJ, Jurrissen TJ, Hannan LC, Zwetsloot KA, Needle AR, et al. Daily watermelon consumption decreases plasma sVCAM-1 levels in overweight and obese postmenopausal women. Nutr Res. 2020;76:9-19.

9. Wong A, Chernykh O, Figueroa A. Chronic l-citrulline supplementation improves cardiac sympathovagal balance in obese postmenopausal women: A preliminary report. Auton Neurosci. 2016;198:50-3.

10. Figueroa A, Alvarez-Alvarado S, Ormsbee MJ, Madzima TA, Campbell JC, Wong A. Impact of L-citrulline supplementation and whole-body vibration training on arterial stiffness and leg muscle function in obese postmenopausal women with high blood pressure. Exp Gerontol. 2015;63:35-40.

11. Wong A, Alvarez-Alvarado S, Jaime SJ, Kinsey AW, Spicer MT, Madzima TA, Figueroa A. Combined whole-body vibration training and l-citrulline supplementation improves pressure wave reflection in obese postmenopausal women. Appl Physiol Nutr Metab. 2016;41(3):292-7.

12. Figueroa A, Wong A, Hooshmand S, Sanchez-Gonzalez MA. Effects of watermelon supplementation on arterial stiffness and wave reflection amplitude in postmenopausal women. Menopause. 2013;20(5):573-7.
